# Comorbidity sequence, sex, and APOE-genotype forecast Alzheimer's disease diagnosis

**DOI:** 10.3389/fmed.2026.1826377

**Published:** 2026-06-11

**Authors:** Simona Merlini, Roberto Gatta, Stefania Orini, Amna Basharat, Muddassar Farooq, Roberta Diaz Brinton, Francesca Vitali

**Affiliations:** 1 Center for Innovation in Brain Science, The University of Arizona, Tucson, AZ, United States; 2 Department of Biomedical Engineering, University of Arizona College of Engineering, Tucson, AZ, United States; 3 Department of Clinical and Experimental Sciences, Universita' degli Studi di Brescia, Brescia, Italy; 4 Cognitive Disorders and Dementia Rehabilitation Unit, Memory Clinic, IRCCS Istituto Centro San Giovanni di Dio Fatebenefratelli, Brescia, Italy; 5 CureMD Research, New York, NY, United States; 6 Department of Neurology, University of Arizona College of Medicine, Tucson, AZ, United States; 7 Department of Pharmacology, University of Arizona College of Medicine, Tucson, AZ, United States; 8 Center for Biomedical Informatics and Biostatistics, University of Arizona, Tucson, AZ, United States

**Keywords:** Alzheimer's disease, APOE genotype, biological sex, precision prevention, process mining, risk factor

## Abstract

**Introduction:**

Alzheimer's disease (AD) is a highly heterogeneous neurodegenerative disorder and the leading cause of dementia characterized by the progressive accumulation of non-modifiable (age, female sex, APOE-ε4 genotype) and modifiable factors [hypertension (HTN), diabetes, obesity (OB), hyperlipidemia (HLP), depression (DEP)]. However, the temporal sequencing and interaction patterns between comorbidity burden and biological subgroups defined by sex and APOE genotype remain not fully understood.

**Methods:**

We applied the Cumulative Event Method (CEM), a novel process mining framework, to longitudinal UK Biobank (UKB) data. Event logs tracked five modifiable risk factors across sex- and APOE-ε4-stratified analyses to identify distinct longitudinal comorbidity patterns associated with AD. Sex-specific findings were validated in an independent CureMD cohort.

**Results:**

Among 1,916 UK Biobank participants, CEM identified 203 distinct comorbidity sequences across 7,316 clinical events. Females more frequently exhibited a hypertension-preceding-AD sequences than males (7.0% vs. 3.8%; *p* = 0.005), while males exhibited earlier metabolic-vascular patterns involving hyperlipidemia and hypertension (7.7% vs. 4.5%; *p* = 0.0085). APOE-ε4 carriers exhibited accelerated multi-comorbidity patterns, whereas non-carriers more frequently transitioned from hypertension to non-AD (*p* = 1 × 10^−4^). External validation in CureMD confirmed sex-specific patterns across 191 sequences and 5,176 events.

**Conclusion:**

Longitudinal comorbidities patterns preceding AD differ by sex and APOE genotype, supporting Alzheimer's as a multisystem failure disease with subgroup-specific comorbidity sequences and clinically relevant windows for precision prevention.

## 1 Introduction

Alzheimer's disease (AD) is a progressive neurodegenerative disorder and the leading cause of dementia worldwide, affecting over 55 million individuals (1). Current treatment approved for AD primarily focus on alleviating the symptoms (2), while recently approved anti-amyloid monoclonal antibodies (1, 3) are considered disease-modifying but pose challenges, including the risk of ARIA/ARIA-E and significant economic burden (1). However, none of these pharmacological therapies reverse disease progression or target upstream risk factors to prevent disease onset.

AD pathogenesis develops over decades and reflects the interplay between non-modifiable factors (1) [age (4, 5), sex (6), APOE genotype (7, 8)] and a broad spectrum of modifiable comorbidities (9, 10), including hypertension (HTN) (11–14), type 2 diabetes (T2D) (15), obesity (OB) (16, 17), hyperlipidemia (HLP) (18, 19), and depression (DEP) (20). While these individual associations are well documented, increasing evidence indicates that AD does not arise from isolated risk factors but from heterogeneous, temporally ordered trajectories of comorbidity accumulation (21–26).

The timing, duration, and sequence of these exposures appear to critically impact AD risk. We previously identified critical age-related tipping points at 62 and 72 years when the impact of risk factors on AD risk significantly increases (27). Specifically, hypertension or diabetes diagnosed before age 62 conferred greater AD risk than APOE-ε4 alone, while obesity diagnosed between ages 62–72 nearly tripled AD risk. These findings indicate that AD progression is driven by dynamic, cumulative pathogenic processes and that both the timing and duration of risk factor exposure critically impact AD development (27). However, a fundamental question remains unresolved: How do multiple comorbidities accumulate and interact over time, and do these accumulation patterns differ by sex and APOE genotype?

Traditional epidemiological approaches often treat comorbidities as static risk factors alone, failing to capture the temporal sequencing, co-occurrence patterns, and cascading effects that characterize real-world disease progression (4, 28–30). Longitudinal health data further complicate analysis: events occur at irregular intervals, individuals differ in follow-up duration, and comorbidity profiles evolve in high-dimensional and non-linear patterns that challenge traditional survival models (31–33). In contrast, process mining techniques, originally developed for business process analysis, offer a promising approach to extract temporal sequences, identify clinical trajectories, and characterize the evolving structure of disease directly from longitudinal health records (34–36).

To overcome limitations of existing methods, we applied the Cumulative Event Method (CEM), a novel process mining analytical framework that captures the temporal sequence and morbidity load of risk factors over time driving AD progression. Prior work has analyzed multi-morbid trajectories preceding dementia using distinct methodological approaches, such as directional-pair analysis on ICD-10 disease pairs in the UK Biobank (UKB) with separate APOE-ε4 stratification (37), Fine-Gray hazard modeling with dynamic-time-warping trajectory clustering in U.S. EHR data (38), and descriptive prevalence ranking of AD comorbidities (39). Our approach uniquely applies a process-mining cumulative-state framework (CEM) that preserves the full ordered history of diagnoses at each temporal level, jointly stratifies by sex and APOE-ε4, and is validated in an independent U.S. EHR cohort. CEM advances traditional techniques by: (1) organizing disease development into data-driven temporal levels, (2) tracking cumulative diagnostic load rather than isolated transitions, and (3) enabling statistical comparison of pathways across biological subgroups. In this study, we apply CEM to reconstruct sex- and genotype-specific temporal trajectories of AD-related comorbidities using UK Biobank longitudinal data, with external sex-specific validation in an independent CureMD cohort.

We hypothesized that: (1) AD progression follows multiple distinct comorbidity accumulation patterns rather than a single common pathway; (2) these pathways differ systematically by sex and APOE-ε4 carrier status; and (3) the resulting patterns reflect underlying vascular and metabolic mechanisms that drive heterogeneous risk across subpopulations.

## 2 Methods

### 2.1 Study design and participants

This study employed a nested case-control design embedded within a retrospective cohort. Cases were defined as UK Biobank (UKB) participants who developed Alzheimer's disease during follow-up, and were matched 1:1 to controls who did not develop AD but were drawn from the same underlying population-based cohort of approximately 500,000 participants aged 40–69 years recruited between 2006 and 2010 across the United Kingdom. From 502,412 UKB participants, we selected individuals aged ≥55 years at recruitment with minimum 3-year follow-up and no prior history of neurodegenerative disease, brain cancer, traumatic brain injury, neurosurgery or early-onset AD (Supplementary Table S1). The age and follow-up inclusion criteria were established to ensure that participants were at risk of developing late-onset AD during the study period, while preserving an adequate follow-up duration to capture disease onset and progression. The 3-year minimum follow-up was chosen to balance data availability with statistical power, while maintaining a sufficient observation window for AD diagnosis. Notably, individuals enrolled at age 55 could be followed for up to 13 years, thereby enabling the assessment of transition to AD. APOE genotype was determined using two key single-nucleotide polymorphisms (SNPs): rs429358 and rs7412. Genotyping was performed using UK BiLEVE Axiom array and UKB Axiom array, with quality control procedures performed centrally by UKB. APOE-ε4 carriers were defined as individuals with at least one ε4 allele (genotypes ε2/ε4, ε3/ε4, or ε4/ε4). Non-carriers had genotypes ε2/ε2, ε2/ε3, or ε3/ε3.

To assess potential confounding by cerebrovascular pathology, we evaluated the presence of diagnostic codes related to cerebrovascular encephalopathy and vascular dementia (ICD-9 codes 437.0, 437.1, 437.3, 437.9. 438.0, 290.40) as well as late effects of cerebrovascular disease (ICD-9 codes 438.89, 438.9). None of the participants in the analyzed UK Biobank cohort carried these specific diagnostic codes; therefore, analyses were not further stratified by AD subtype.

External validation was conducted using data from CureMD, a U.S.-based multisite electronic health record system serving over 30,000 providers across multiple states. The CureMD cohort included a total of 4,449,857 participants and was used as independent validation of sex-specific findings. APOE genotyping data were not available and genotype-stratified analyses could not be replicated in this independent dataset.

To ensure comparability between AD cases and controls, we implemented 1:1 propensity score matching on both cohorts using the *MatchIt* R package. Matching variables included baseline age, follow-up duration, recruitment center, education level, and Charlson Comorbidity Index (CCI). CCI was calculated excluding the five modifiable risk factors of interest (hypertension, type 2 diabetes, obesity, hyperlipidemia, depression) to avoid conditioning on exposures of interest.

### 2.2 Alzheimer's disease diagnosis

Analysis focused exclusively on late-onset AD diagnoses, identified via ICD-10 diagnostic codes (F00, G30, F03 with contributory AD) recorded during life or listed as primary/contributory cause of death (Supplementary Table S2). For AD diagnoses recorded at death, introducing uncertainty in AD onset timing, linear regression model using participants with lifetime-recorded diagnoses was used to estimate onset timing for post-mortem cases. This approach was applied to 144 participants (14.3% of AD cases), as reported in Table 1. Post-mortem cases were retained to avoid systematic exclusion of potentially more severe disease presentations, which could bias comorbidity trajectory reconstruction.

**Table 1:** Baseline demographic and clinical characteristics of UK Biobank participants with and without Alzheimer's disease (AD) after 1:1 propensity score matching.

Variable	Non-AD (*N* = 958)	AD (*N* = 958)	*p*-value
Sex			
Female	495 (51.7%)	522 (54.5%)	0.216
Male	463 (48.3%)	436 (45.5%)	
Age			
Mean (*SD*)	65.346 (3.311)	65.333 (3.301)	0.934
Range	55.000–70.000	55.000–70.000	
APOE			
E4 non-carrier	731 (76.3%)	376 (39.2%)	<0.001
E4 carrier	227 (23.7%)	582 (60.8%)	
Comorbidities			
Diabetes	213 (22.2%)	252 (26.3%)	<0.001
Hypertension	802 (83.7%)	762 (79.5%)	<0.001
Hyperlipidemia	394 (41.1%)	415 (43.3%)	<0.001
Obesity	176 (18.4%)	96 (10.0%)	<0.001
Depression	107 (11.2%)	189 (19.7%)	<0.001
Follow-up time [years]			
3–5	25 (2.5%)	18 (1.9%)	>0.99
6–10	256 (26.8%)	269 (28.1%)	
11–15	677 (70.7%)	671(70%)	
Comorbidity diagnosis age [years]			
Depression			
Mean (*SD*)	71.916 (5.498)	71.910 (5.500)	>0.99
Range	57.000–82.000	55.000–83.000	
Diabetes			
Mean (*SD*)	71.075 (7.079)	69.036 (7.564)	0.003
Range	51.000–83.000	45.000–83.000	
Hypertension			
Mean (*SD*)	70.663 (6.574)	69.144 (6.844)	<0.001
Range	47.000–84.000	50.000–85.000	
Obesity			
Mean (*SD*)	72.795 (5.959)	71.302 (7.195)	0.068
Range	57.000–84.000	51.000–83.000	
Hyperlipidemia			
Mean (*SD*)	71.701 (6.315)	69.961 (6.658)	<0.001
Range	51.000–84.000	49.000–83.000	
Alzheimer's			
Mean (*SD*)		75.971 (4.160)	
Range		59.000–85.000	
Death register data			
#death	76 (7.5%)	388 (38.5%)	<0.001
Death for Alzheimer's		144 (14.3%)	
Age at death			
Mean (*SD*)	78.042 (3.454)	76.703 (4.065)	0.01
Range	68.000–84.000	61.000–84.000	

Groups were matched on age at recruitment, sex, education level, recruitment center, Charlson comorbidity index (CCI), and years of follow-up. Values are reported as *n* (%) or mean (SD). *p*-values reflect between-group comparisons after matching.

### 2.3 Comorbidity definitions

Clinical diagnoses for five major modifiable AD risk factors were extracted using ICD-10-CM codes for both cohorts (Supplementary Table S2). For UKB, data were extracted from primary care records (Data Field 41202) and hospital inpatient records (Data Field 41270). All comorbidity diagnoses were time-stamped to enable temporal sequence analysis. For individuals diagnosed with AD, only risk factor diagnoses occurring before AD onset were included in trajectory analysis. For controls, all diagnoses up to the last recorded clinical contact were included. Although study enrollment was restricted to individuals aged 55 years and older, prior exposure to AD risk factors was fully captured through UKB's comprehensive linked electronic health records, which integrate hospital inpatient and primary care data, enabling retrospective assessment of risk factor trajectories preceding recruitment.

### 2.4 Process mining approach

The Cumulative Event Method (CEM) is a novel process mining analytical framework designed to capture temporal sequences and cumulative diagnostic burden in disease progression (34, 36). CEM advances beyond traditional process mining approaches by: (1) temporal stratification into discrete data-driven levels based on cumulative number of diagnoses; (2) tracking of previous states to maintain complete history of prior diagnoses, enabling tracking of multi-step progressions, (3) enabling statistical comparison of pathway frequency differences between biological subgroups. CEM can be applied by varying 3 different parameters: (1) threshold, minimum frequency for edge inclusion; (2) min.abs, minimum absolute count for node retention; and (3) clusterNum, number of major progression clusters. For the UK Biobank cohort, CEM parameters were set as follows: threshold = 50, min.abs = 50, and clusterNum = 4. For the CureMD validation cohort, given its different sample size and data density, parameters were set to threshold = 20, min.abs = 20, and clusterNum = 4. These values were selected to ensure that only clinically meaningful and statistically robust transitions were retained in the trajectory graph, while avoiding the “spaghetti effect” (4), a well-recognized phenomenon in process mining whereby overly permissive thresholds produce densely connected, uninterpretable graphs. Importantly, each level in the CEM graph does not simply represent the next sequential diagnosis, but groups together all clinical events occurring within a specific data-driven time window, defined by the distribution of inter-event times across all participants. Transitions between successive levels therefore reflect progression through distinct temporal periods of the disease course, with earlier levels corresponding to diagnoses occurring further from AD onset and later levels reflecting diagnoses occurring closer in time to AD. The number of time windows is determined by the clusterNum parameter (set to 4 in this analysis). CEM workflow is available in Supplementary Materials.

For this analysis, event logs were extracted to obtain a matrix of transition *T* where each row represented a timestamped clinical event (diagnosis) for each participant. The matrix *T* included: (1) Participant ID, (2) Event type (diagnosis of HTN, T2D, OB, HLP, DEP, AD, or no AD for last follow-up), (3) Event timestamp (date at diagnosis), (4) Sex and APOE genotype (meta-attributes). Define for each row *k*:


$$\begin{array}{l} T_{k}=\;\left(ID_{k},\;Event_{k}\;,\;Time_{k}\;,\;Sex_{k}\;,\;APOE_{k}\right),\;k\;=\;1,\ldots ,M. \end{array}$$

Where rows are ordered temporally by (*ID, t*). Events included diagnoses of hypertension (HTN), type 2 diabetes (T2D), obesity (OB), hyperlipidemia (HLP), depression (DEP), AD, or end of follow-up without AD.

To ensure comparable timeframes across participants, all trajectories were aligned to a baseline age of 37 years, this threshold was selected as 37yo was the earliest age with a recorded diagnosis in the cohort. Participants with only a single clinical event (i.e., no comorbidity diagnosis among the five modifiable risk factors of interest before AD or before the end of follow-up) were excluded from trajectory analysis as they provided insufficient information for comorbidity progression pattern analysis. Of the 1,411 AD participants eligible after initial screening, 453 (32.1%) were excluded on this basis.

### 2.5 Statistical analysis

Participant characteristics were summarized using descriptive statistics. Categorical variables were reported as frequencies and percentages; continuous variables as means and standard deviations. Differences between cases and controls were assessed using χ^2^ tests for categorical variables and *t*-tests for continuous variables.

CEM-derived comorbidity sequences were compared across sex and APOE genotype (carriers defined as having at least one ε4 allele vs. non-carriers) using Fisher exact tests for proportions. All analyses were performed using R version 4.2.0. Process mining analyses were conducted using the pMineR package (40), freely available at https://github.com/PMLiquidLab/pMineR.v046.

## 3 Results

### 3.1 Cohort characteristics

**Figure 1:**
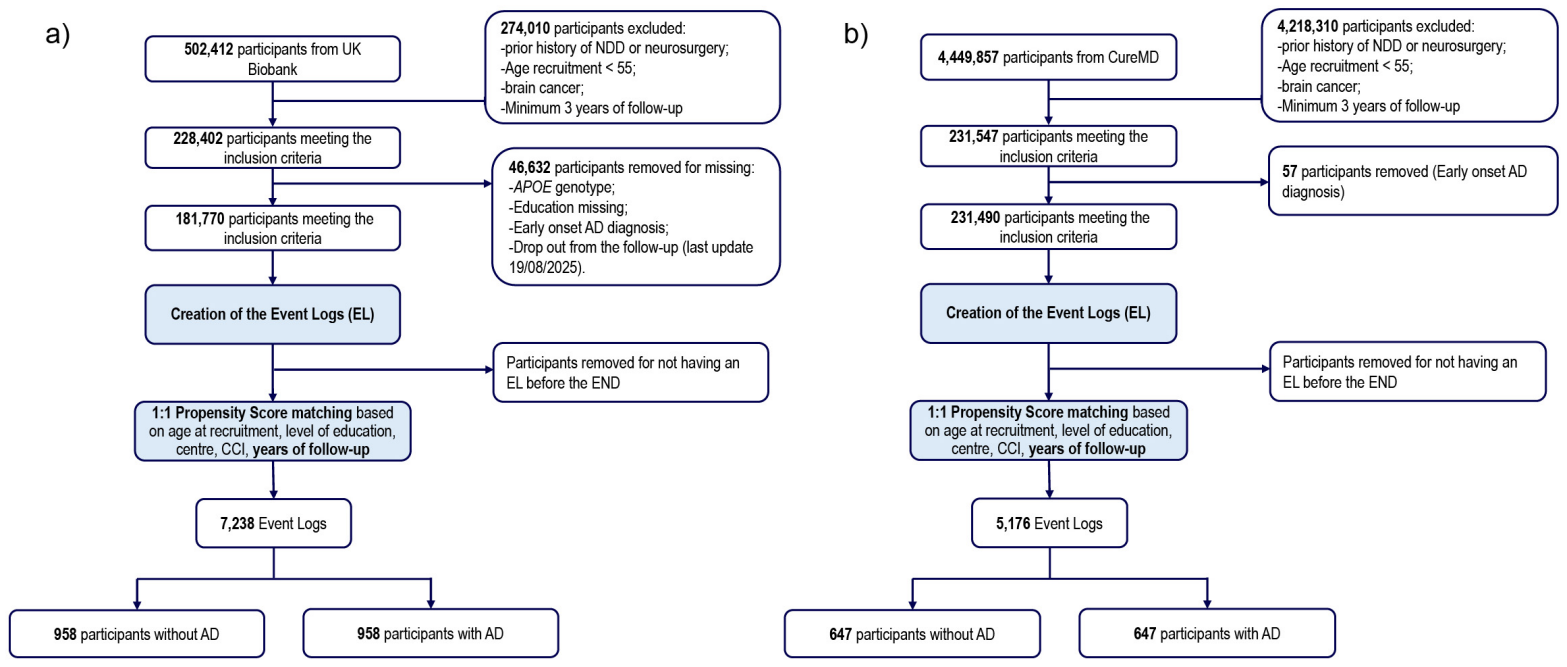
Study design of the **(a)** UK Biobank (UKB) cohort and **(b)** CureMD cohort. Flow diagrams illustrate participant inclusion and exclusion criteria, event log (EL) creation, and 1:1 propensity score matching based on age at recruitment, education level, recruitment center, Charlson Comorbidity Index (CCI), and years of follow-up, resulting in matched cohorts with and without Alzheimer's disease (AD) in the two datasets.

From 502,412 UK Biobank participants, we identified 1,916 individuals (958 matched pairs) meeting inclusion criteria after 1:1 propensity score matching (Figure 1a). The matched cohort had a mean recruitment age of 65.3 ± 3.3 years and mean AD diagnosis age of 75.97 ± 4.16 years. APOE-ε4 carriers included 42.2% (809/1,916) of the cohort. Sex distribution was 53.1% female (1,017/1,916) and 46.9% male (899/1,916). After matching, no significant differences were observed in baseline age, follow-up duration, education level, or Charlson Comorbidity Index between cases and controls (Table 1). APOE-ε4 carrier status differed significantly between AD cases (60.8%) and controls (23.7%, *p* < 0.001), confirming its role as a major genetic risk factor (Table 1). Additional plots illustrating the distribution of demographic and baseline characteristics in the UKB matched cohort are available in the Supplementary Materials (Supplementary Figure S1 and Supplementary Table S3).

### 3.2 Comorbidity sequences in UK Biobank

CEM algorithm applied to the matched UKB cohort identified 203 distinct patterns of risk factor trajectories across 7,316 clinical events, revealing a complex heterogeneity underlying AD progression. The corresponding event-to-event transition matrices summarizing these patterns are provided in the Supplementary Materials (Supplementary Table S4).

Analysis of the most frequent progression patterns revealed dominance of trajectories including hypertension (Table 2). Hypertension appeared in 7 of the top 10 progression patterns, accounting for 974 of 1,155 participants (84.3%) represented in these common trajectories. The most common pattern was HTN → No AD (*n* = 345, 18.0% of cohort), followed closely by HTN → AD (*n* = 279, 14.6%), representing direct progression from hypertension to AD without intermediate comorbidities.

**Table 2:** Top 10 most frequent longitudinal comorbidity progression patterns identified in the UK Biobank (UKB) and CureMD cohorts.

Pathways (UKB)	*F* (*n*)	*P* (%)	Pathways (CureMD)	*F* (*n*)	*P* (%)
Start → HTN → no AD	345	36	Start → HTN → AD	156	24.1
Start → HTN → AD	279	29.1	Start → HLP → HTN → no AD	131	20.2
Start → HLP → HTN → AD	97	10.1	Start → HTN → No AD	128	19.8
Start → HLP → HTN → no AD	74	7.7	Start → HLP → AD	97	15
Start → HTN → HLP → no AD	71	7.4	Start → HLP → HTN → AD	73	11.3
Start → HTN → HLP → AD	69	7.2	Start → T2D → no AD	58	9
Start → HLP → AD	68	7.1	Start → HLP → HTN → T2D → no AD	47	7.3
Start → HLP → no AD	60	6.3	Start → HLP → no AD	46	7.1
Start → DEP → AD	53	5.5	Start → HTN → T2D → AD	38	5.9
Start → T2D → HTN → AD	39	4.1	Start → HLP → HTN → T2D → AD	37	5.7

Patterns represent ordered sequences of diagnoses from baseline (“Start”) to Alzheimer's disease (AD) or non-AD status. Frequency indicates the number of participants exhibiting each sequence; prevalence is calculated as the percentage of AD and non-AD cases in each cohort. AD, Alzheimer's disease; HLP, hyperlipidemia; HTN, hypertension; T2D, type 2 diabetes; DEP, depression; P, prevalence; F, frequency.

Multi-comorbidity vascular progression patterns were also highly prevalent. HLP → HTN → AD pathway occurred in 97 participants (5.1%), while its non-AD equivalent (HLP → HTN → No AD) occurred in 74 participants (3.9%). Notably, the reverse comorbidity sequence HTN → HLP → No AD (*n* = 71, 3.7%) and HTN → HLP → AD (*n* = 69, 3.6%) exhibited nearly equivalent frequencies. The equivalent AD frequency suggests that the co-occurrence of vascular risk factors may be more critical than their temporal order of diagnosis (Supplementary Figure S2). This equivalence is visible across all levels of the CEM diagram (Supplementary Figure S2). At level 1, participants split into HTN-first (*n* = 306/1,916) and HLP-first (*n* = 55/1,916) trajectories, and this pattern persists across subsequent levels, where both the HLP → HTN and HTN → HLP sequences converge toward AD, suggesting that the presence of both conditions, rather than their diagnostic order, drives AD risk (Supplementary Figure S2).

**Figure 2:**
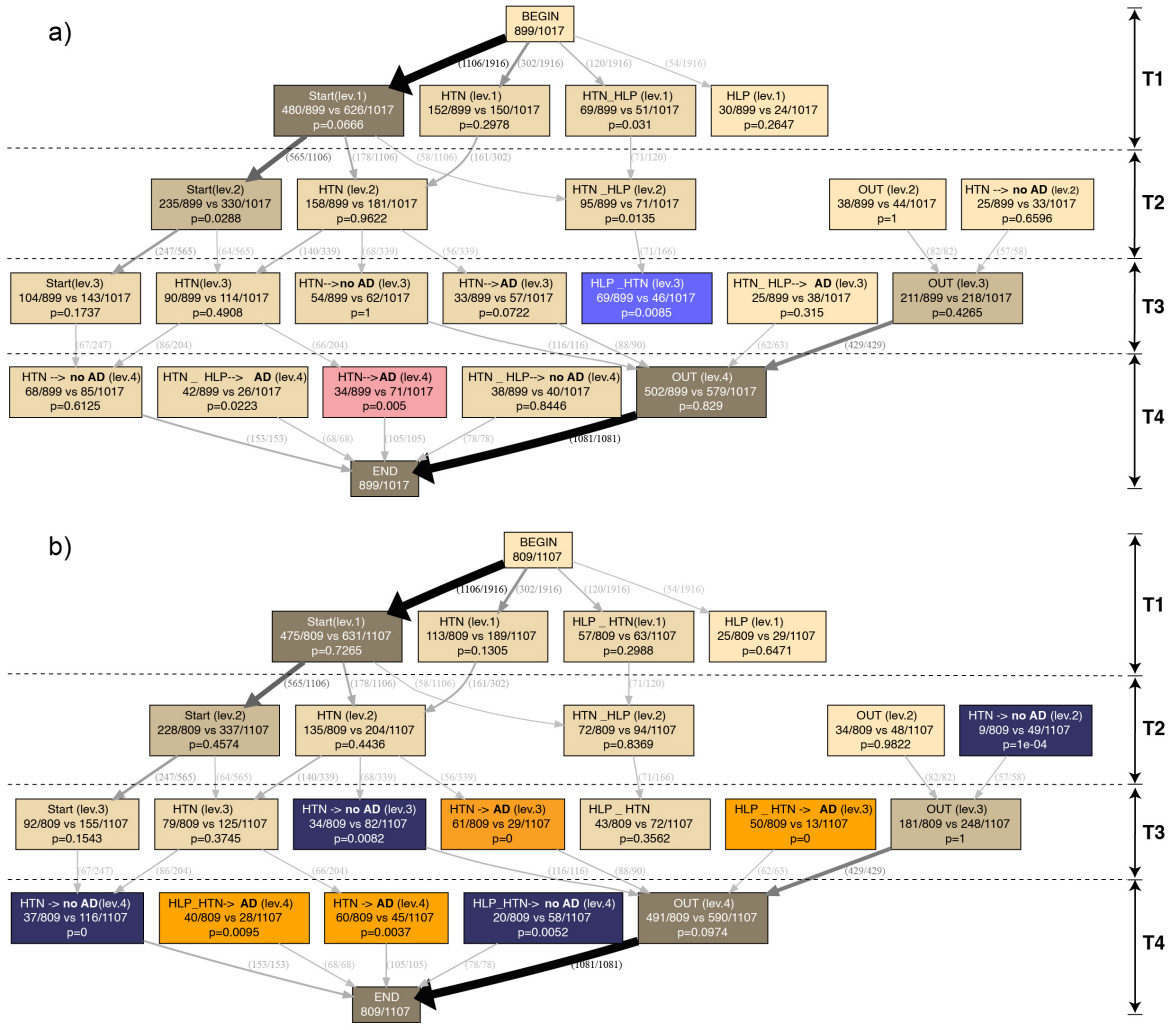
Sex- and APOE-ε4-stratified comorbidity progression trajectories in Alzheimer's disease (AD) using cumulative event modeling (CEM) on 1,916 UK Biobank participants. Nodes represent cumulative comorbidity states, with edges indicating temporal progression patterns (threshold = 50, min.abs = 50, clusterNum = 4). **(a)** CEM with sex stratification. Blue nodes indicate pathways significantly overrepresented in males; pink nodes indicate pathways significantly overrepresented in females (*p* < 0.05, chi-square test). The direct pathway hypertension-to-AD (HTN → AD) occurred significantly more frequently in females compared to males. **(b)** CEM with APOE genotype stratification. Orange nodes indicate pathways significantly overrepresented in APOE-ε4 carriers; blue nodes indicate pathways significantly overrepresented in non-carriers (*p* < 0.05, chi-square test). APOE-ε4 carriers exhibited significantly more direct hypertension-to-AD (HTN → AD) pathways and multi-factorial progression routes (HTN + HLP → AD) compared to non-carriers, with genetic differences persisting across progression levels. Temporal window labels T1–T4, displayed on the right-hand side of each panel, denote the four data-driven time windows into which the CEM algorithm partitions the follow-up period based on the distribution of inter-event times across all participants. T1 corresponds to the earliest temporal window from study entry, while T4 corresponds to the window closest to AD diagnosis or end of follow-up, reflecting the most proximal period of comorbidity accumulation preceding the terminal event. Abbreviations HTN, hypertension; HLP, hyperlipidemia; AD, Alzheimer's disease.

Supplementary Table S5 reports the full set of sex-stratified node-level counts and χ^2^ tests underlying Figure 2a; Supplementary Table S6 provides the corresponding APOE-ε4-stratified results underlying Figure 2b.

### 3.3 Sex-specific comorbidity patterns

Sex-stratified CEM analysis revealed significantly different comorbidity progression patterns between females (*n* = 1,017) and males (*n* = 899; Figure 2a). Females demonstrated a significantly higher frequency of hypertension-preceding-AD diagnostic sequence (HTN → AD: 71/1,017, 7.0%) compared to males (34/899, 3.8%; *p* = 0.005). Conversely, males exhibited significantly greater sequential metabolic-vascular comorbidity patterns, with the HLP+HTN sequence occurring in 7.7% of males (69/899, 7.7%) vs. 4.5% of females (46/1,017, 4.5%; *p* = 0.0085; Figure 2a).

### 3.4 APOE genotype-specific patterns

APOE-ε4 genotype stratification (ε4 carriers: *n* = 809; non-carriers: *n* = 1,107) revealed significantly different progression patterns linked to genetic susceptibility (Figure 2b). APOE-ε4 carriers demonstrated significantly higher rates of multi-factorial progression pathways involving sequential risk factor accumulation. The HTN + HLP → AD pathway occurred significantly more frequently in APOE-ε4 carriers (50/809, 6.1%) compared to non-carriers (13/1,107, 1.17%; *p* < 0.001). This genetic difference persisted across progression levels, with carriers exhibiting enrichment at later levels (carrier: 4.9% vs. non-carrier: 2.5%, *p* = 0.0095, level 3 and level 4). The direct HTN → AD pathway was also more prevalent in APOE-ε4 carriers vs. non-carriers at level 3 (carrier: 7.5% vs. non-carrier: 2.6%; *p* < 0.001) and level 4 (carrier: 7.5% vs. non-carrier: 4%, *p* = 0.0037). Conversely, APOE-ε4 non-carriers exhibited significantly higher rates of hypertension without progression to AD at earlier level (HTN → No AD: 49/1,107, 4.4%) compared to carriers (9/809, 1.1%; *p* < 0.001) suggesting greater resilience to hypertension-driven neurodegeneration in the absence of genetic risk.

### 3.5 External validation using CureMD medical records

Independent validation in the propensity score-matched CureMD cohort (*n* = 1,294; 813 females, 481 males; mean age 79.4 ± 8.4 years; 647 AD cases, Figure 1b, Supplementary Figure S3) identified 191 distinct patterns across 5,176 clinical events (Table 1, Supplementary Table S7). Remarkably, the top 2 progression patterns in CureMD closely mirrored UKB findings. The most common CureMD pattern was HTN → AD (*n* = 156, 12.5%), consistent with UKB where this ranked as the second most common pattern (14.6%; Table 2). The sequential vascular progression HLP → HTN → No AD ranked second in CureMD (*n* = 131, 10.5%), compared to 3.9% frequency in UKB (Table 2).

**Figure 3:**
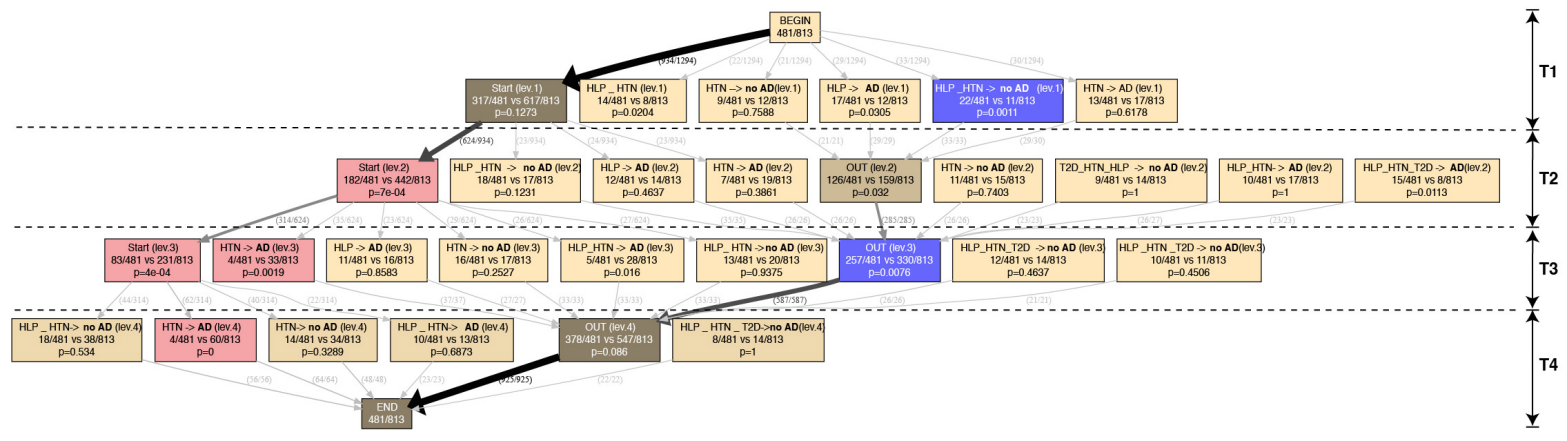
Sex-stratified comorbidity progression trajectories in Alzheimer's disease using CEM analysis on 1,294 CureMD patients. Nodes represent cumulative comorbidity states, with edges indicating temporal progression patterns (threshold = 20, min.abs = 20, clusterNum = 4). Blue nodes indicate pathways significantly overrepresented in males; pink nodes indicate pathways significantly overrepresented in females (*p* < 0.05, chi-square test). The hypertension-preceding-AD diagnosis sequence (HTN → AD) was observed significantly more frequently in females than in males. Temporal window labels T1–T4, displayed on the right-hand side of each panel, denote the four data-driven time windows into which the CEM algorithm partitions the follow-up period based on the distribution of inter-event times across all participants. T1 corresponds to the earliest temporal window from study entry, while T4 corresponds to the window closest to AD diagnosis or end of follow-up, reflecting the most proximal period of comorbidity accumulation preceding the terminal event. Abbreviations HTN, hypertension; HLP, hyperlipidemia; AD, Alzheimer's disease.

Sex-stratified CureMD analysis confirmed the key patterns identified in UK Biobank (Figure 3). The direct HTN → AD pathway was significantly more common in females (4.0%) compared to males (0.8%, *p* = 0.0019), consistent with UKB findings. Similarly, the sequential HLP + HTN progression was more frequent in males (4.5%) vs. females (1.3%, *p* = 0.0011), replicating the pattern observed in UKB. This independent validation in a demographically distinct US healthcare cohort strengthens the generalizability of sex-specific progression patterns. Supplementary Table S8 reports the full set of sex-stratified node-level counts and χ^2^ tests underlying Figure 3.

## 4 Discussion

Our analyses revealed that AD progression follows distinct temporal trajectories of risk factor accumulation that differ systematically by sex and APOE genotype. Using the novel CEM framework applied to 1,916 UK Biobank participants, we identified sex-specific patterns, validated in an independent CureMD cohort and characterized by direct hypertension-to-AD progression in females vs. early metabolic-vascular patterns in males, and genotype-specific patterns demonstrating accelerated multi-factorial convergence in APOE-ε4 carriers. These findings establish Alzheimer's as a multisystem failure disease with biological subgroup-specific temporal signatures.

Our sex specific findings align with established sex differences in cardiovascular aging and cerebrovascular regulation (41). Postmenopausal loss of estrogen's protective effects increases vascular stiffness and blood pressure variability, potentially explaining why late-onset hypertension in females more directly impacts cerebrovascular integrity and AD risk (41–43). The observation that females in our cohort developed hypertension at temporal levels closer to AD diagnosis than males further supported this temporal relationship. Conversely, the earlier metabolic-vascular patterns in males may reflects sex differences in adipose tissue distribution, insulin sensitivity, and inflammatory profiles. Males develop central adiposity and metabolic syndrome at younger ages, potentially triggering earlier vascular dysfunction through mechanisms including endothelial dysfunction, oxidative stress, and chronic inflammation (41, 44).

Analyses of CEM including APOE genotype revealed that APOE-ε4 carriers were associated with accelerated progression to AD, as evidenced by their higher frequency of multi-comorbidity patterns at earlier temporal levels of the CEM graph, reflecting a more compressed time window of cumulative diagnostic accumulation preceding AD onset. This aligns with APOE-4's pleiotropic effects on lipid metabolism (45), vascular function (46), and amyloid clearance (47). APOE-ε4 carriers are associated with impaired cerebrovascular reactivity and blood-brain barrier integrity even before amyloid accumulation, potentially explaining their heightened vulnerability to vascular risk factors (47, 48).

The enrichment of hypertension and hyperlipidemia preceding AD in APOE-ε4 carriers suggests genetic predisposition increases susceptibility to cumulative risk factor burden overwhelming compensatory mechanisms (49). This supports our previous finding that risk factor duration exceeds APOE-ε4 impact at younger ages, while APOE-ε4 becomes dominant after age 72 when multisystem resilience declines (27). Conversely, the higher rate of hypertension-to-no AD patterns in ε4 non-carriers indicates a greater compensatory capacity. This finding suggests that non-carriers, despite vascular dysregulation caused by hypertension, are more resilient and effectively maintain cerebrovascular autoregulation and neurovascular coupling (50).

The interplay between temporal progression patterns and genetic susceptibility suggests that previously identified age tipping points might also depend on the burden of accumulated risk factors, reflecting individual-specific cumulative vulnerability. This supports a dynamic model where AD risk emerges from interactions between temporal risk accumulation patterns, biological sex, and genetic predisposition.

Our findings broadly align with prior work identifying cardiometabolic conditions as dominant precursors of dementia. Both Fu et al. (38) and Li et al. (37) report cardiovascular- and circulatory-initiated trajectories leading to AD, and Rogers et al. (39) highlights hypertension and hyperlipidaemia as among the most prevalent AD comorbidities. Our joint sex and APOE-ε4 stratification extends these observations by showing that the same cardiometabolic dysfunction can produce distinct sequences depending on biological subgroup, most notably a direct HTN → AD pathway in female APOE-ε4 carriers.

Findings from this study provide evidence to support precision AD prevention strategies and their implications are highly translational. First, the identified progression patterns suggest sex-specific screening and intervention protocols for AD risk factors, where females may benefit from intensive monitoring and management of late-life hypertension, while males warrant earlier metabolic-vascular risk assessment and intervention. Second, the accelerated risk progression of APOE-ε4 carriers influenced by multiple risk factors underlines the need of heightened surveillance and earlier risk factor management strategies. Third, collectively the identified progression patterns support precision intervention strategies that can be tailored to sex-, genotype-, and pathway-specific interventions. For example, males with an earlier diagnosis of hyperlipidemia resulted in higher risk for AD when hypertension was subsequently diagnosed. Therefore, this group might benefit from enhanced hypertension monitoring and prevention.

Collectively, these results strongly support AD as a multisystem failure disease rather than a single-pathway condition. The identification of 203 distinct progression patterns across 7,316 events demonstrates heterogeneity extending far beyond accounting for risk factors alone. This breadth of variation highlights that AD-related comorbidity accumulation does not follow a single dominant trajectory; rather, individuals traverse highly individualized pathways that differ in the number, type, and sequence of diagnoses. These findings align with emerging concepts of biological aging as multisystem decline (51) and suggests AD prevention requires coordinated management of interconnected metabolic, vascular, and inflammatory systems rather than isolated treatment of individual conditions.

Our application of CEM advances methodological approaches to understanding disease progression. Unlike traditional survival models or cross-sectional risk factor analyses, CEM framework offers several advantages over traditional approaches: (1) preservation of temporal information lost in cross-sectional or time-aggregated analyses; (2) representation and visualization of complex multi-step comorbidity sequences; and (3) statistical comparison of pathway frequencies between subgroups. However, our threshold-based approach (threshold = 50) underrepresented lower-frequency comorbidities including depression, diabetes, and obesity in complex pathways. Future applications could employ adaptive thresholding or complementary analyses to capture clinically meaningful but less prevalent patterns.

Our cohort limitations include UK Biobank's limited ethnic diversity, potentially restricting generalizability to non-European populations. The CureMD validation cohort, while confirming sex-specific patterns, had no information regarding APOE genotyping, preventing full replication of genotype-stratified findings. In addition, the relatively small sample size may be viewed as a limitation. However, this characteristic is also an asset as distinct progression patterns were detected and replicated in an independent dataset of comparable size. This methodology supports the power of the CEM analytical approach for healthcare systems serving smaller populations typically found in rural locations. Reliance on ICD-based diagnostic codes in both datasets may underrepresent subclinical disease or underdiagnosed conditions influenced by healthcare utilization patterns. To address this concern in the UK Biobank, we evaluated the presence of diagnostic codes related to cerebrovascular encephalopathy and vascular dementia as well as late effects of cerebrovascular disease. Within the UK Biobank cohort analyzed in this study, none of the participants carried these specific codes, supporting that our findings primarily reflect individuals diagnosed with non-cerebrovascular AD pathology. Future studies leveraging on large-scale datasets incorporating biomarker data, such as blood-based biomarkers, cerebrospinal fluid (CSF), positron emission tomography (PET) imaging, will be critical to validate these findings in pathologically confirmed AD cases.

Several issues warrant further investigation. First, incorporation of treatment data (antihypertensives, statins, diabetes medications) would reveal whether specific therapeutic interventions modify progression trajectories. Second, extension to additional comorbidities (e.g., atrial fibrillation, chronic kidney disease, sleep disorders) could identify broader multimorbidity networks driving neurodegeneration. Third, integration with biomarker data could link clinical progression patterns to underlying amyloid, tau, and neuroinflammatory trajectories. Fourth, application of CEM to other neurodegenerative diseases (Parkinson's disease, frontotemporal dementia, vascular dementia) could reveal shared vs. disease-specific progression signatures. Finally, development of individualized risk prediction tools incorporating both baseline characteristics and real-time trajectory monitoring could enable dynamic risk stratification and personalized prevention strategies.

Longitudinal patterns of comorbidity diagnoses preceding AD differ systematically by sex and APOE genotype. These findings support the concept of AD as a multisystem failure disease characterized by subgroup-specific comorbidity sequences rather than a single common pathway. The identification of distinct temporal trajectories demonstrates that trajectory-based analytical approaches can identify clinically relevant windows for precision prevention strategies targeting at-risk populations. Future research should investigate biological mechanisms underlying these sex- and genotype-specific trajectories and test whether personalized prevention strategies based on trajectory profiles can reduce AD incidence.

## 5 Conclusions

This study demonstrates that Alzheimer's disease progression is characterized by heterogeneous and temporally ordered comorbidity trajectories that differ systematically according to biological sex and APOE-ε4 genotype. Using the novel CEM applied to longitudinal UK Biobank data and independently validated in the CureMD cohort, we identified reproducible sex- and genotype-specific patterns of vascular and metabolic risk accumulation preceding AD diagnosis.

Collectively, these findings support the concept of AD as a multisystem failure disease rather than a disorder driven by a single pathogenic pathway. Importantly, our results highlight clinically relevant windows for precision prevention and suggest that trajectory-based risk profiling may improve early identification of high-risk individuals and inform sex- and genotype-tailored prevention strategies. Future studies integrating biomarkers, treatment exposure, and additional comorbidities will be essential to further refine individualized prediction models and determine whether targeted intervention along specific comorbidity trajectories can delay or prevent AD onset.

## Data Availability

The original contributions presented in the study are included in the article/Supplementary material, further inquiries can be directed to the corresponding author.
