# Correction: Comorbidity sequence, sex, and APOE-genotype forecast Alzheimer's disease diagnosis

**DOI:** 10.3389/fmed.2026.1947497

**Published:** 2026-08-05

**Authors:** Simona Merlini, Roberto Gatta, Stefania Orini, Amna Basharat, Muddassar Farooq, Roberta Diaz Brinton, Francesca Vitali

**Affiliations:** 1 Center for Innovation in Brain Science, The University of Arizona, Tucson, AZ, United States; 2 Department of Biomedical Engineering, University of Arizona College of Engineering, Tucson, AZ, United States; 3 Department of Clinical and Experimental Sciences, Università degli Studi di Brescia, Brescia, Italy; 4 Cognitive Disorders and Dementia Rehabilitation Unit, Memory Clinic, IRCCS Istituto Centro San Giovanni di Dio Fatebenefratelli, Brescia, Italy; 5 CureMD Research, New York, NY, United States; 6 Department of Neurology, University of Arizona College of Medicine, Tucson, AZ, United States; 7 Department of Pharmacology, University of Arizona College of Medicine, Tucson, AZ, United States; 8 Center for Biomedical Informatics and Biostatistics, University of Arizona, Tucson, AZ, United States

**Keywords:** Alzheimer's disease, APOE genotype, biological sex, precision prevention, process mining, risk factor

The **affiliation** “Department of Clinical and Experimental Sciences, Università degli Studi di Brescia, Brescia, Italy” was mistakenly listed for author Stefania Orini. This affiliation has been removed for this author.

The original version of this article has been updated.

