# Supplementary material for: Comorbidity sequence, sex, and APOE-genotype forecast Alzheimer's disease diagnosis
Source: Front Med (Lausanne). 2026 Jun 11;13:1826377. doi: 10.3389/fmed.2026.1826377 (PMC13293945; doi:10.3389/fmed.2026.1826377)
Supplement: Supplementary file 1 [file Supplementary_file_1.docx]

Supplementary Table S1. ICD-10-CM code used for exclusion criteria.

| **Disease** | **ICD codes** |
| --- | --- |
| Neurological Disorders / Brain Neoplasms | C71.7, C71.8, C71.9, C79.31, D33.0, D33.1, D33.2, D43.0, D43.1, D43.2,D49.6, G93.1, G93.5, G93.82 |
| Traumatic Brain Injury (TBI) | S06.317A, S06.317S, S06.327A, S06.337A, S06.337S, S06.377A, S06.380A, S06.380D, S06.380S, S06.381A, S06.381D, S06.382S, S06.384A, S06.385S, S06.387A, S06.387S, S06.389A, S06.389D, S06.389S |
| Infectious Brain Diseases | A06.6, A17.81, A54.82, B43.1 |
| History of Brain Neoplasms | Z85.841, Z86.011 |

Supplementary Table S2. ICD-10-CM code used for clinical diagnosis of Alzheimer's and related modifiable risk factors.

| **Disease** | **ICD codes** |
| --- | --- |
| Diabetes | E08, E09, E10, E11, E13 |
| Hypertension | I10, I11, I12, I13, I14, I15 |
| Hyperlipidemia | E78 |
| Obesity | E66 |
| Depression | F32, F33 |
| Alzheimer’s | G30, G301, G308, G309 |


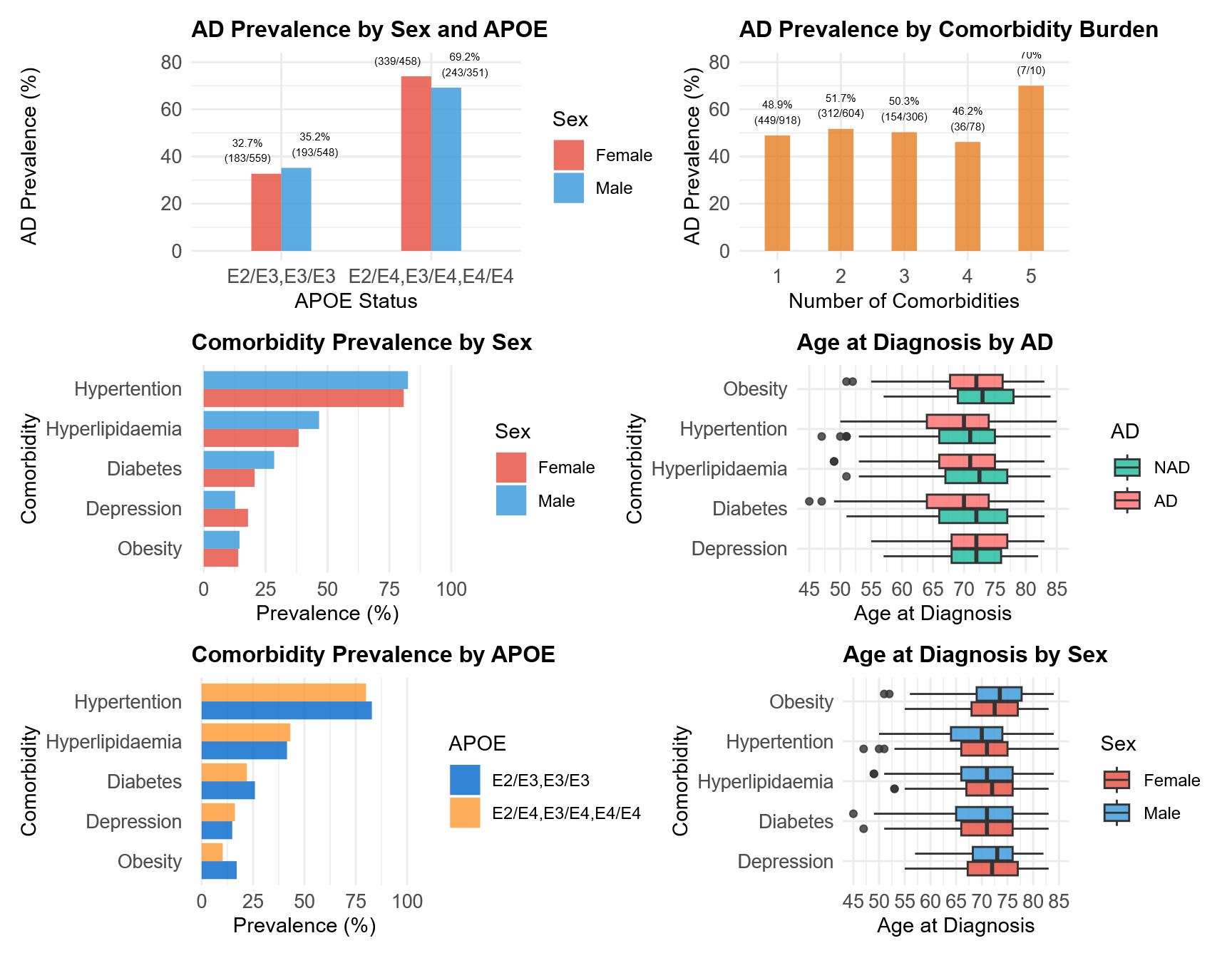


**Supplementary Figure S1:** Main characteristics of the UK Biobank matched cohort.

**Supplementary Table S3:** Baseline demographic and clinical characteristics of UK Biobank participants with and without Alzheimer’s disease (AD) before 1:1 propensity score matching. P values reflect between-group comparisons.

|  | **Non-AD** | **AD** |  |
| --- | --- | --- | --- |
|  | **(N=180,300)** | **(N=1,411)** | p value |
| Sex | | | |
| Female | 101173 (56.1%) | 796 (56.4%) | 0.821 |
| Male | 79127 (43.9%) | 615 (43.6%) |  |
| Age | | | |
| Mean (SD) | 61.727 (4.061) | 65.128 (3.359) | < 0.001 |
| Range | 55.000 - 73.000 | 55.000 - 70.000 |  |
| APOE | | | |
| E4 noncarrier | 132926 (73.7%) | 524 (37.1%) | < 0.001 |
| E4 carrier | 47374 (26.3%) | 887 (62.9%) |  |
| Comorbidities | | | |
| Diabetes | 16000 (8.9%) | 278 (19.7%) | < 0.001 |
| Hypertension | 62657 (34.8%) | 815 (57.8%) | < 0.001 |
| Hyperlipidemia | 32453 (18.0%) | 461 (32.7%) | < 0.001 |
| Obesity | 11865 (6.6%) | 108 (7.7%) | 0.105 |
| Depression | 8793 (4.9%) | 189 (13.4%) | < 0.001 |
| Follow-up time [years] | | | |
| 0-2 | 45601 (25.2%) | 9 (0.7%) | < 0.001 |
| 3-5 | 10961 (6.1%) | 32 (2.3%) |  |
| 6-10 | 58181 (32.3%) | 416 (29.5%) |  |
| 11-15 | 65557 (36.4%) | 954(67.6%) |  |
| Comorbidity diagnosis age [years] | | | |
| Depression | | | |
| Mean (SD) | 67.613 (7.034) | 71.910 (5.500) | < 0.001 |
| Range | 43.000 - 83.000 | 55.000 - 83.000 |  |
| Diabetes | | | |
| Mean (SD) | 66.986 (6.880) | 68.839 (7.520) | < 0.001 |
| Range | 43.000 - 85.000 | 45.000 - 83.000 |  |
| Hypertension | | | |
| Mean (SD) | 66.647 (6.806) | 68.734 (6.797) | < 0.001 |
| Range | 43.000 - 84.000 | 50.000 - 84.000 |  |
| Obesity | | | |
| Mean (SD) | 63.895 (5.572) | 67.137 (5.271) | < 0.001 |
| Range | 44.000 - 83.000 | 50.000 - 83.000 |  |
| Hyperlipidemia | | | |
| Mean (SD) | 67.440 (6.745) | 69.647 (6.622) | < 0.001 |
| Range | 42.000 - 84.000 | 48.000 - 82.000 |  |
| Alzheimer's | | | |
| Mean (SD) |  | 75.045 (4.374) |  |
| Range |  | 54.000 - 84.000 |  |
| Death Register Data | | | |
| #death | 8023 (4.4%) | 569 (40.3%) | < 0.001 |
| Death for Alzheimer’s |  | 235 (16.7%) |  |
| Age at Death | | | |
| Mean (SD) | 72.428 (5.472) | 76.009 (4.167) | < 0.001 |
| Range | 55.7- 84.3 | 60.5 – 84.00 |  |


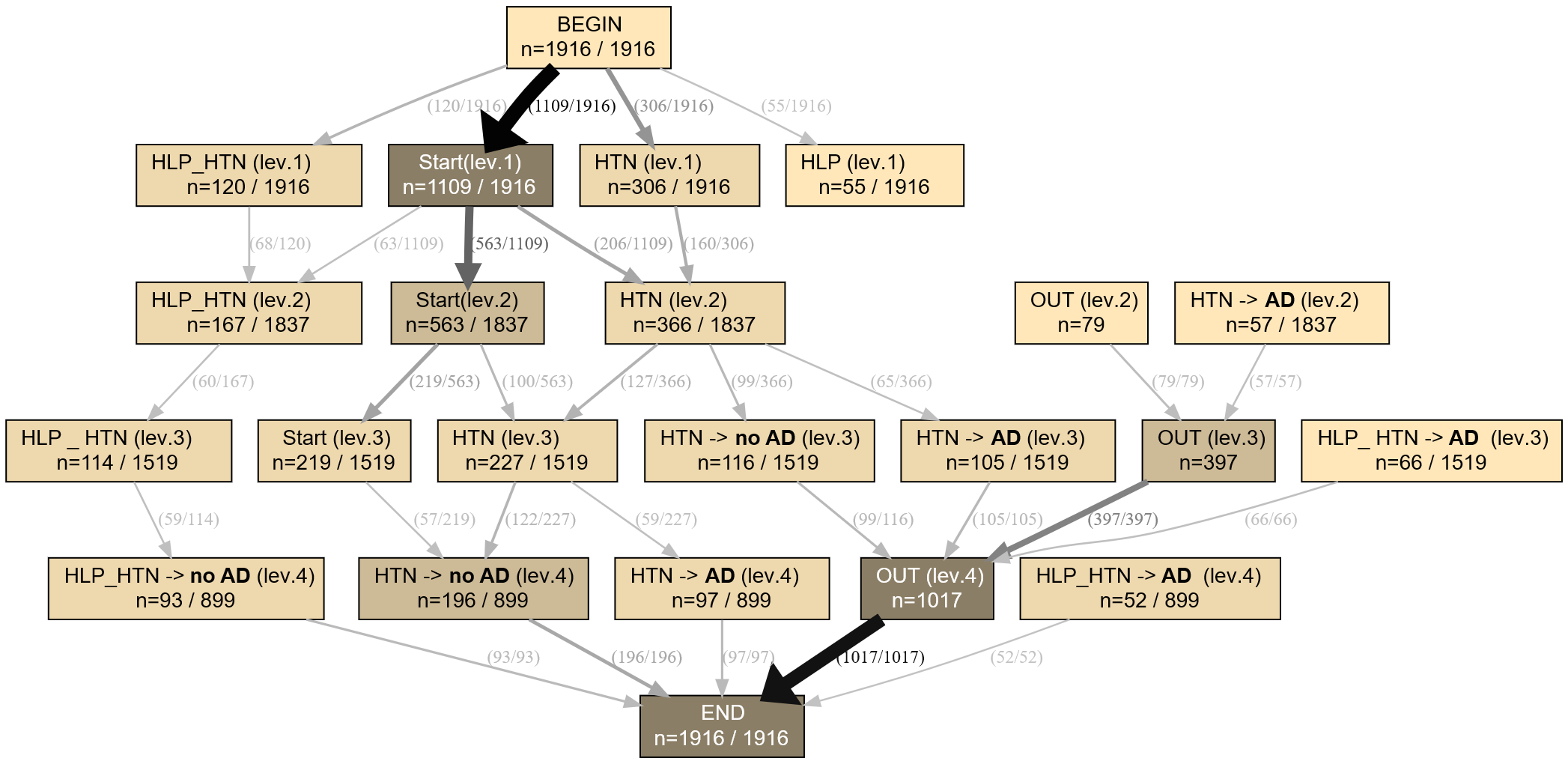


**Supplementary Figure S2**: *Comorbidity progression trajectories in Alzheimer’s Disease (AD) using Cumulative Event Modelling (CEM) on 1,916 UK Biobank participants.* The diagram illustrates ordered diagnosis events and transitions across successive levels from baseline (“BEGIN”) to study exit (“END”) among 1,916 participants. Nodes represent comorbidity states, edges denote observed transitions, and labels indicate the number of individuals in each state at each level. AD and non-AD outcomes are highlighted as terminal absorbing states. The equivalence in AD frequency between the HLP→HTN and HTN→HLP trajectories visible from level 1 onwards, where participants diverge based on which vascular risk factor was diagnosed first and their convergence toward comparable terminal AD frequencies across all subsequent levels, suggests that the co-occurrence of these vascular risk factors may be more critical than their temporal order of diagnosis.

**Supplementary Table S4:** UK Biobank Transition Matrix. This table represents a first-order Markov model of comorbidity progression, where each cell reports the number of participants (and the corresponding row percentage in parentheses) who transitioned from the state listed in the row to the state listed in the column. Each row therefore sums to 100% across all possible subsequent states, representing the conditional probability of transitioning to each next state given the current state. For example, of all participants in the HTN state, 31% subsequently transitioned to HLP, 17.3% to T2D, and 33.1% to NAD (end of follow-up without AD diagnosis). The BEGIN row indicates the initial state of all 1,916 participants entering the model, while the AD and NAD rows represent absorbing terminal states, from which no further transitions occur. Abbreviations: AD, Alzheimer's disease; NAD, no Alzheimer's disease; OB, obesity; DEP, depression; HLP, hyperlipidemia; HTN, hypertension; T2D, type 2 diabetes.

|  | **END** | **Start** | **HTN** | **AD** | **HLP** | **T2D** | **OB** | **DEP** | **NAD** |
| --- | --- | --- | --- | --- | --- | --- | --- | --- | --- |
| **BEGIN** | 0 | 1916(100) | 0 | 0 | 0 | 0 | 0 | 0 | 0 |
| **END** | 0 | 0 | 0 | 0 | 0 | 0 | 0 | 0 | 0 |
| **Start** | 0 | 0 | 1009 (52.6) | 0 | 437 (22.8) | 248 (12.9) | 56 (2.9) | 166 (8.6) | 0 |
| **HTN** | 0 | 0 | 0 | 485 (31) | 271 (17.3) | 97 (6.2) | 116 (7.4) | 77 (4.9) | 518 (33.1) |
| **AD** | 958 (100) | 0 | 0 | 0 | 0 | 0 | 0 | 0 | 0 |
| **HLP** | 0 | 0 | 257 (31.7) | 193 (23.8) | 0 | 90 (11.1) | 58 (7.2) | 27 (3.3) | 184 (22.7) |
| **T2D** | 0 | 0 | 208(44.7) | 100 (21.5) | 39 (8.4) | 0 | 26 (5.6) | 10 (2.1) | 82 (17.6) |
| **OB** | 0 | 0 | 24 (8.8) | 66 (24.3) | 23 (8.4) | 15 (5.5) | 0 | 16 (5.8) | 128 (47) |
| **DEP** | 0 | 0 | 66 (22.3) | 114 (38.5) | 39 (13.2) | 15 (5) | 16 (5.4) | 0 | 46 (15.5) |
| **NAD** | 958 (100) | 0 | 0 | 0 | 0 | 0 | 0 | 0 | 0 |

**Supplementary Table S5:** Distribution of males (N=899) and females (N=1017) across all nodes of the decision tree (**Figure 3**). Each row corresponds to a terminal or intermediate node at a given hierarchical level (0–4), corresponding to time periods T1 through T4. Percentages are computed relative to the total number of males or females at BEGIN (N=899 and N=1017, respectively). p-values are from Fisher’s exact test or χ² test comparing males and females proportions at each node. Abbreviations: AD, Alzheimer's disease; NAD, no Alzheimer's disease; HLP, hyperlipidemia; HTN, hypertension.

| **Level** | **Node Label** | **Males (n)** | **Males %** | **Females (n)** | **Females %** | **p-value** |
| --- | --- | --- | --- | --- | --- | --- |
| 0 | **BEGIN** |  |  |  |  |  |
| 1 | Start (lev.1) | 480 | 53.4% | 626 | 61.6% | 0.0666 |
| 1 | HTN (lev.1) | 152 | 16.9% | 150 | 14.7% | 0.2978 |
| 1 | **HTN_HLP (lev.1)** | 69 | 7.7% | 51 | 5.0% | 0.0310 |
| 1 | HLP (lev.1) | 30 | 3.3% | 24 | 2.4% | 0.2647 |
| 2 | **Start (lev.2)** | 235 | 26.1% | 330 | 32.4% | 0.0288 |
| 2 | HTN (lev.2) | 158 | 17.6% | 181 | 17.8% | 0.9622 |
| 2 | **HTN_HLP (lev.2)** | 95 | 10.6% | 71 | 7.0% | 0.0135 |
| 2 | OUT (lev.2) | 38 | 4.2% | 44 | 4.3% | 1.0000 |
| 2 | HTN → no AD (lev.2) | 25 | 2.8% | 33 | 3.2% | 0.6596 |
| 3 | Start (lev.3) | 104 | 11.6% | 143 | 14.1% | 0.1737 |
| 3 | HTN (lev.3) | 90 | 10.0% | 114 | 11.2% | 0.4908 |
| 3 | HTN → no AD (lev.3) | 54 | 6.0% | 62 | 6.1% | 1.0000 |
| 3 | HTN → AD (lev.3) | 33 | 3.7% | 57 | 5.6% | 0.0722 |
| 3 | **HLP_HTN (lev.3)** | 69 | 7.7% | 46 | 4.5% | 0.0085 |
| 3 | HTN_HLP → AD (lev.3) | 25 | 2.8% | 38 | 3.7% | 0.3150 |
| 3 | OUT (lev.3) | 211 | 23.5% | 218 | 21.4% | 0.4265 |
| 4 | HTN → no AD (lev.4) | 68 | 7.6% | 85 | 8.4% | 0.6125 |
| 4 | **HTN_HLP → AD (lev.4)** | 42 | 4.7% | 26 | 2.6% | 0.0223 |
| 4 | **HTN → AD (lev.4)** | 34 | 3.8% | 71 | 7.0% | 0.0050 |
| 4 | HTN_HLP → no AD (lev.4) | 38 | 4.2% | 40 | 3.9% | 0.8446 |
| 4 | OUT (lev.4) | 502 | 55.8% | 579 | 56.9% | 0.8290 |
| 0 | **END** |  |  |  |  |  |

**Supplementary Table S6:** Distribution of APOE4 (N=809) and non APOE4 (N=1107) across all nodes of the decision tree (Figure 2b). Each row corresponds to a terminal or intermediate node at a given hierarchical level (0–4), corresponding to time periods T1 through T4. Percentages are computed relative to the total number of APOE4 or non APOE4 at BEGIN. p-values are from Fisher’s exact test or χ² test comparing APOE4 and non APOE4 proportions at each node. Abbreviations: AD, Alzheimer's disease; NAD, no Alzheimer's disease; HLP, hyperlipidemia; HTN, hypertension.

| **Level** | **Node Label** | **APOE4 (n)** | **APOE4 %** | **non APOE4 (n)** | **non APOE4 %** | **p-value** |
| --- | --- | --- | --- | --- | --- | --- |
| 0 | **BEGIN** |  |  |  |  |  |
| 1 | Start (lev.1) | 475 | 58.7% | 631 | 57.0% | 0.7265 |
| 1 | HTN (lev.1) | 113 | 14.0% | 189 | 17.1% | 0.1305 |
| 1 | HLP_HTN (lev.1) | 57 | 7.0% | 63 | 5.7% | 0.2988 |
| 1 | HLP (lev.1) | 25 | 3.1% | 29 | 2.6% | 0.6471 |
| 2 | Start (lev.2) | 228 | 28.2% | 337 | 30.4% | 0.4574 |
| 2 | HTN (lev.2) | 135 | 16.7% | 204 | 18.4% | 0.4436 |
| 2 | HTN_HLP (lev.2) | 72 | 8.9% | 94 | 8.5% | 0.8369 |
| 2 | OUT (lev.2) | 34 | 4.2% | 48 | 4.3% | 0.9822 |
| 2 | **HTN → no AD (lev.2)** | 9 | 1.1% | 49 | 4.4% | **0.0001** |
| 3 | Start (lev.3) | 92 | 11.4% | 155 | 14.0% | 0.1543 |
| 3 | HTN (lev.3) | 79 | 9.8% | 125 | 11.3% | 0.3745 |
| 3 | **HTN → no AD (lev.3)** | 34 | 4.2% | 82 | 7.4% | 0.0082 |
| 3 | **HTN → AD (lev.3)** | 61 | 7.5% | 29 | 2.6% | **0.0000** |
| 3 | HLP_HTN (lev.3) | 43 | 5.3% | 72 | 6.5% | 0.3562 |
| 3 | **HLP_HTN → AD (lev.3)** | 50 | 6.2% | 13 | 1.2% | **0.0000** |
| 3 | OUT (lev.3) | 181 | 22.4% | 248 | 22.4% | 1.0000 |
| 4 | **HTN → no AD (lev.4)** | 37 | 4.6% | 116 | 10.5% | **0.0000** |
| 4 | **HLP_HTN → AD (lev.4)** | 40 | 4.9% | 28 | 2.5% | 0.0095 |
| 4 | **HTN → AD (lev.4)** | 60 | 7.4% | 45 | 4.1% | 0.0037 |
| 4 | **HLP_HTN → no AD (lev.4)** | 20 | 2.5% | 58 | 5.2% | 0.0052 |
| 4 | OUT (lev.4) | 491 | 60.7% | 590 | 53.3% | 0.0974 |
| 0 | **END** |  |  |  |  |  |


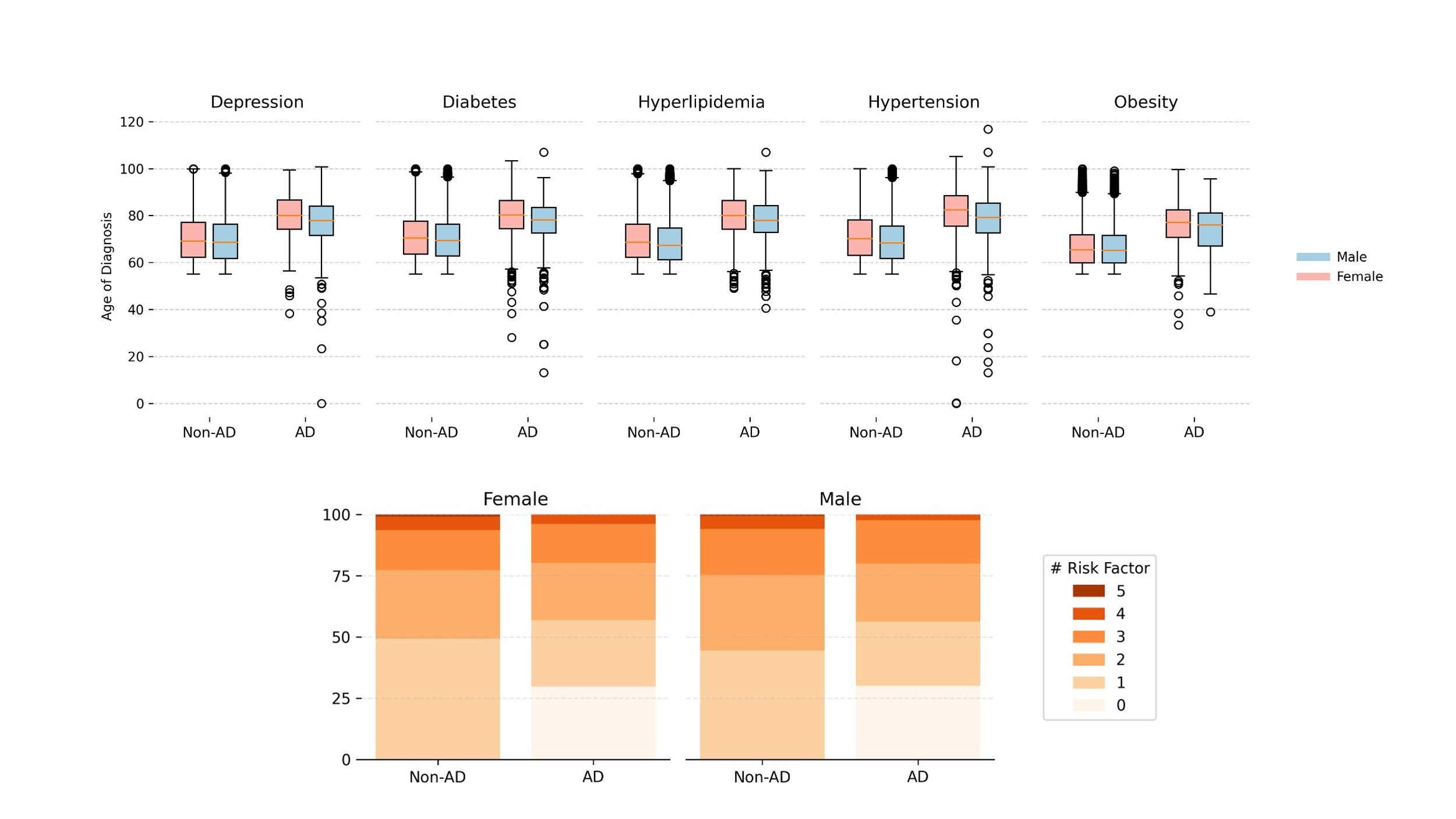


**Supplementary Figure S3:** Main characteristics of the CureMD matched cohort.

**Supplementary Table S7:** CureMD Transition Matrix. This table represents a first-order Markov model of comorbidity progression, where each cell reports the number of participants (and the corresponding row percentage in parentheses) who transitioned from the state listed in the row to the state listed in the column. Each row therefore sums to 100% across all possible subsequent states, representing the conditional probability of transitioning to each next state given the current state. Abbreviations: AD, Alzheimer’s disease; NAD, no Alzheimer’s disease; OB, obesity; DEP, depression; HLP, hyperlipidemia; HTN, hypertension; T2D, type 2 diabetes.

|  | **END** | **Start** | **T2D** | **DEP** | **HLP** | **HTN** | **OB** | **AD** | **NAD** |
| --- | --- | --- | --- | --- | --- | --- | --- | --- | --- |
| **BEGIN** | 0 | 1294 (100%) | 0 (0.0%) | 0 (0.0%) | 0 (0.0%) | 0 (0.0%) | 0 (0.0%) | 0 (0.0%) | 0 (0.0%) |
| **END** | 0 | 0 (0%) | 0 (0.0%) | 0 (0.0%) | 0 (0.0%) | 0 (0.0%) | 0 (0.0%) | 0 (0.0%) | 0 (0.0%) |
| **Start** | 0 | 0 (0%) | 135 (10.4%) | 89 (6.9%) | 539 (41.7%) | 469 (36.2%) | 62 (4.8%) | 0 (0.0%) | 0 (0.0%) |
| **T2D** | 0 | 0 (0%) | 0 (0.0%) | 7 (1.6%) | 51 (11.9%) | 44 (10.3%) | 11 (2.6%) | 142 (33.3%) | 172 (40.3%) |
| **DEP** | 0 | 0 (0%) | 4 (2.6%) | 0 (0.0%) | 46 (29.5%) | 23 (14.7%) | 7 (4.5%) | 47 (30.1%) | 29 (18.6%) |
| **HLP** | 0 | 0 (0%) | 84 (11.3%) | 18 (2.4%) | 0 (0.0%) | 378 (50.9%) | 8 (1.1%) | 158 (21.3%) | 96 (12.9%) |
| **HTN** | 0 | 0 (0%) | 199 (21.1%) | 40 (4.2%) | 78 (8.3%) | 0 (0.0%) | 11 (1.2%) | 289 (30.6%) | 326 (34.6%) |
| **OB** | 0 | 0 (0%) | 5 (5.1%) | 2 (2.0%) | 28 (28.3%) | 29 (29.3%) | 0 (0.0%) | 11 (11.1%) | 24 (24.2%) |
| **AD** | 647 | 0 (0%) | 0 (0.0%) | 0 (0.0%) | 0 (0.0%) | 0 (0.0%) | 0 (0.0%) | 0 (0.0%) | 0 (0.0%) |
| **NAD** | 647 | 0 (0%) | 0 (0.0%) | 0 (0.0%) | 0 (0.0%) | 0 (0.0%) | 0 (0.0%) | 0 (0.0%) | 0 (0.0%) |

**Supplementary Table S8:** Distribution of males (N=481) and females (N=813) across all nodes of the decision tree (**Figure 3**). Each row corresponds to a terminal or intermediate node at a given hierarchical level (0-4), corresponding to time periods T1 through T4. Percentages are computed relative to the total number of males or females at BEGIN (N=481 and N=813, respectively). p-values are from Fisher’s exact test or χ² test comparing males and females proportions at each node. Abbreviations: AD, Alzheimer's disease; NAD, no Alzheimer's disease; HLP, hyperlipidemia; HTN, hypertension.

| **Level** | **Node Label** | **Males (n)** | **Males %** | **Females (n)** | **Females %** | **p-value** |
| --- | --- | --- | --- | --- | --- | --- |
| 1 | Start (lev.1) | 317 | 65.9% | 617 | 75.9% | 0.1273 |
| 1 | **HLP_HTN → no AD (lev.1)** | 14 | 2.9% | 8 | 1.0% | **0.0204** |
| 1 | HTN → no AD (lev.1) | 9 | 1.9% | 12 | 1.5% | 0.7588 |
| 1 | **HLP → AD (lev.1)** | 17 | 3.5% | 12 | 1.5% | **0.0305** |
| 1 | **HLP_HTN → no AD (lev.1)** | 22 | 4.6% | 11 | 1.4% | **0.0011** |
| 1 | HTN → AD (lev.1) | 13 | 2.7% | 17 | 2.1% | 0.6178 |
| 2 | Start (lev.2) | 182 | 37.8% | 442 | 54.4% | **7×10⁻⁴** |
| 2 | HLP_HTN → no AD (lev.2) | 18 | 3.7% | 17 | 2.1% | 0.1231 |
| 2 | HLP → AD (lev.2) | 12 | 2.5% | 14 | 1.7% | 0.4637 |
| 2 | HTN → AD (lev.2) | 7 | 1.5% | 19 | 2.3% | 0.3861 |
| 2 | OUT (lev.2) | 126 | 26.2% | 159 | 19.6% | **0.032** |
| 2 | HTN → no AD (lev.2) | 11 | 2.3% | 15 | 1.8% | 0.7403 |
| 2 | T2D_HTN_HLP → no AD (lev.2) | 9 | 1.9% | 14 | 1.7% | 1 |
| 2 | HLP_HTN → AD (lev.2) | 10 | 2.1% | 17 | 2.1% | 1 |
| 2 | **HLP_HTN_T2D → AD (lev.2)** | 15 | 3.1% | 8 | 1.0% | **0.0113** |
| 3 | Start (lev.3) | 83 | 17.3% | 231 | 28.4% | **4×10⁻⁴** |
| 3 | **HTN → AD (lev.3)** | 4 | 0.8% | 33 | 4.1% | **0.0019** |
| 3 | HLP → AD (lev.3) | 11 | 2.3% | 16 | 2.0% | 0.8583 |
| 3 | HTN → no AD (lev.3) | 16 | 3.3% | 17 | 2.1% | 0.2527 |
| 3 | **HLP_HTN → AD (lev.3)** | 5 | 1.0% | 28 | 3.4% | **0.016** |
| 3 | HLP_HTN → no AD (lev.3) | 13 | 2.7% | 20 | 2.5% | 0.9375 |
| **3** | **OUT (lev.3)** | 257 | 53.4% | 330 | 40.6% | **0.0076** |
| 3 | HLP_HTN_T2D → no AD (lev.3) | 12 | 2.5% | 11 | 1.4% | 0.4637 |
| 3 | HLP_HTN_T2D → no AD (lev.3) | 10 | 2.1% | 11 | 1.4% | 0.4506 |
| 4 | HLP_HTN → no AD (lev.4) | 18 | 3.7% | 38 | 4.7% | 0.534 |
| 4 | **HTN → AD (lev.4)** | 4 | 0.8% | 60 | 7.4% | **<0.001** |
| 4 | HTN → no AD (lev.4) | 14 | 2.9% | 34 | 4.2% | 0.3289 |
| 4 | HLP_HTN → AD (lev.4) | 10 | 2.1% | 13 | 1.6% | 0.6873 |
| 4 | OUT (lev.4) | 378 | 78.6% | 547 | 67.3% | **0.086** |
| 4 | HLP_HTN_T2D → no AD (lev.4) | 8 | 1.7% | 14 | 1.7% | 1 |

# **Supplementary Methods: Cumulative Event Modeling (CEM) Workflow**

Cumulative Event Modeling (CEM) was implemented as a process-mining analytical framework to reconstruct and compare longitudinal multimorbidity trajectories while preserving temporal ordering and cumulative disease history.

**Input Data**. For each individual i, longitudinal diagnosis records were extracted as time-stamped events and ordered chronologically across the observation period.

**Temporal partitioning.** The follow-up time was partitioned into four data-driven temporal windows (T1-T4), defined consistently across individuals within each cohort. These windows capture successive phases of disease accumulation prior to the outcome of interest.

**Cumulative state construction.** Within each temporal window, newly observed diagnoses were added to the existing disease history, generating a sequence of cumulative states for each individual. Thus, each state represents the full set of diagnoses observed up to and including that time window.

Formally, for individual i, the cumulative disease state at window $T_{k},$ is defined as:

$$S_{i}(T_{k}) = S_{i}(T_{\{k-1\}})\cup E_{i}(T_{k}),$$

where $E_{i}(T_{k}),$ denotes the set of diagnoses first observed within window $T_{k}$ and $S_{i}(T_{k})$represents the accumulated diagnosis history up to that time.

**Transition extraction**. Transitions between consecutive cumulative states ($S_{i}(T_{k})$→$S_{i}$ ($T_{\{k+1\}}$)) were computed for each individual. These transitions encode the progression of multimorbidity over time.
